# Practical Anatomy

**Published:** 1900-07

**Authors:** 


Practical Anatomy Including a Special Section on the Fundamental Principles of Anatomy. Edited by W. T. Eckley, M. D., Professor of Anatomy in the College of Physicians and Surgeons, University of Illinois; Professor of Anatomy in the Northwestern University Dental School; Professor of Anatomy in the Chicago Clinical School, and Director of the Chicago School of Anatomy and Physiology; Member of the American Medical Association, the Chicago Pathological Society, the Chicago Medical Society, etc.; and Mrs. Corinne Duford Eckley, Instructor of Anatomy in the Northwestern University Woman's Medical School; Professor of Anatomy in the Chicago School of Anatomy and Physiology. With 347 illustrations, many of which are in colors. Octavo. Philadelphia: P Blakiston's Son & Co., 1012 Walnut Street. 1899 [Price, cloth, $3.50 net; oil cloth, $4.00 net.]

This work will be found a great help to the beginner in the study of anatomy for the authors have blended together, in a practical manner, anatomical structures as found in the dissecting-room with little quizes that go a long way toward elucidating the subject. The book is profusely illustrated and stress is laid on the relations between the somatic and sympathetic nerves in a way quite new to the usual treatise on anatomy. A dissecting-room guide to any descriptive anatomy is always a help to the student and in this work is found a valuable introduction and guide to Gerrish's Descriptive Anatomy, lately published. As a quiz compend on anatomy and a guide to dissection it fills a useful place. M. C.

